# Development of High-Production Bacterial Biomimetic Vesicles for Inducing Mucosal Immunity Against Avian Pathogenic *Escherichia coli*

**DOI:** 10.3390/ijms252212055

**Published:** 2024-11-09

**Authors:** Yue Li, Yuji Quan, Peng Chen, Xiangkai Zhuge, Tao Qin, Sujuan Chen, Daxin Peng, Xiufan Liu

**Affiliations:** 1 College of Veterinary Medicine, Yangzhou University, Jiangsu Co-Innovation Center for the Prevention and Control of Important Animal Infectious Disease and Zoonoses, Yangzhou 225009, China; dz120200015@yzu.edu.cn (Y.L.); mz120231753@stu.yzu.edu.cn (Y.Q.); mz120231662@stu.yzu.edu.cn (P.C.); qintao@yzu.edu.cn (T.Q.); xfliu@yzu.edu.cn (X.L.); 2 Joint International Research Laboratory of Agriculture and Agri-Product Safety, The Ministry of Education of China, Yangzhou 225009, China; 3 Jiangsu Research Centre of Engineering and Technology for Prevention and Control of Poultry Disease, Yangzhou 225009, China; 4 School of Public Health, Nantong University, Nantong 226019, China; zhugexk@ntu.edu.cn

**Keywords:** avian pathogenic *Escherichia coli*, outer membrane vesicles, bacterial biomimetic vesicles, intranasal immunization

## Abstract

To evaluate the immunoprotective effect of bacterial biomimetic vesicles (BBVs) against avian pathogenic *Escherichia coli* (APEC), a Δ*tolA* J11 mutant strain was generated by deleting the *tolA* gene in the low pathogenic O78 serotype J11 strain. The total protein content of outer membrane vesicles (OMVs) derived from the Δ*tolA* J11 strain exhibited a sevenfold increase compared to the wild-type strain. Additionally, high-pressure homogenization technology was employed to produce BBVs, resulting in a sixfold increase in total protein content compared to spontaneously secreted OMVs from Δ*tolA* J11. The immunogenicity of both OMVs and BBVs was assessed through intranasal or intramuscular immunization in specific pathogen-free (SPF) chickens. Results demonstrated that intranasal immunization with OMVs or BBVs in chickens elicited specific IgY antibodies against APEC outer membrane proteins and specific sIgA antibodies in the nasal cavity and trachea, as well as a significant increase in the proliferation response of chicken peripheral blood lymphocytes. The bacterial load in the blood and various organs of the challenged chickens were significantly reduced, resulting in a 66.67% and 58.30% survival rate against a high pathogenic serotype O78 strain challenge, while the control group exhibited only a 16.67% survival rate. The intramuscular immunization with OMVs or BBVs in chickens only induced specific IgY antibodies, with a survival rate of only 33.33% for challenged chickens during the same period. Therefore, intranasal vaccination of the highly productive BBVs is capable of eliciting an immune response similar to that of OMVs and providing protection against APEC infection, thus offering innovative insights for the advancement of APEC vaccines.

## 1. Introduction

Avian pathogenic *Escherichia coli* (APEC) is one of the most common bacterial pathogens in poultry. Among the many APEC serotypes, O78 is a prevalent serotype [1,2]. Although APEC may persist in the digestive tract of healthy chickens, the respiratory tract is considered an important infection route, leading to a diverse array of infections ranging from local to systemic conditions, such as air sacculitis, perihepatitis, pericarditis, oviductitis, and peritonitis, culminating in acute septicemia and fatalities [3]. This poses significant economic burdens on the global poultry industry [4]. The control of APEC infections has traditionally relied on antibiotics; however, the extensive utilization of antibiotics has resulted in the emergence of severe resistance among APEC strains [5,6]. Alarmingly, a study conducted in eastern China revealed that 66.1% of APEC isolates exhibited resistance to ultra-broad-spectrum cephalosporins [7]. Of utmost concern is the overlap between antimicrobial drugs prescribed for humans and animals, as antibiotic-resistant *E. coli* not only compromises treatment efficacy but also poses a substantial threat to human public health and safety [8].

In addition to management and husbandry practices, vaccination is an important method of preventing and controlling APEC by activating the host immune response to eliminate bacteria from the body [9]. Currently, the main vaccine types against APEC include inactivated, subunit, and live attenuated vaccines. However, each type possesses its own set of limitations [10]. Inactivated vaccines are not widely employed due to their limited protection against homologous challenges only. Additionally, their protective effect is influenced by various factors, such as the adjuvant used, the route of administration, and the age of the poultry [4]. Subunit vaccines provide wider protection against multiple serotypes of APEC. Nevertheless, they have been documented to cause stress to birds during vaccination and may have side effects associated with strong adjuvants [11]. At present, the licensed live attenuated APEC O78 Δ*aroA* vaccine (Poulvac^®^ *E. coli*) provides clinical protection against the challenge in homologous intra-airsac. However, Poulvac^®^ *E. coli* strain can be recovered from the lungs and internal organs of vaccinated turkeys, which increases the risk of vaccine spreading and acquisition of virulent genes from other *E. coli*. And the “alerta inmune” state produced by a vaccine may be related to weight loss [12,13,14]. Given these limitations, there is a pressing need to continue researching novel vaccine candidates against APEC to provide safe and effective protection for poultry.

In recent years, nanovaccines have garnered increasing attention as vaccine platforms against bacterial pathogens. Outer membrane vesicles (OMVs) secreted by Gram-negative bacteria are natural, non-replicable spherical nanoparticles, comprising outer membrane proteins (Omps), lipids, periplasmic proteins, polysaccharides, and nucleic acids [15]. OMVs present an attractive bacterial vaccine candidate owing to their excellent intrinsic immunostimulatory properties [16,17]. Traditional inactivated vaccines and subunit vaccines typically denature proteins and other components during the production process, thereby reducing the antigenic diversity of the vaccine. OMVs maintain an antigen library in their native conformation, potentially offering broader and more effective protection [18]. Actually, the 4CMenB OMV vaccine [Bexsero, GSK] for Neisseria meningitidis serogroup B (MenB) has been approved for use in 40 countries. 4CMenB contains three antigenic components, fHbp, NHBA, and NadA, which provide extensive protection for invasive MenB strains [19,20]. In recent years, more and more reports have proved that OMVs have great potential in enhancing the immune system. Intranasal immunization with *Acinetobacter baumannii* OMVs reduced the airway colonization and provided strong immune protection [21]. Subcutaneous injection of BSA-OMV nanoparticles against carbapenem-resistant *Klebsiella pneumoniae* (CRKP) induced humoral and cellular immunity, which significantly improved the survival rate of mice infected with a lethal dose of CRKP [22]. However, few studies pay attention to the mucosal immune protection of bacterial vesicles against APEC. Furthermore, the quantity of OMVs spontaneously released by wild-type *E. coli* is insufficient to facilitate large-scale production for candidate vaccines [17]. Therefore, it is necessary to develop other methods to increase the number of vesicles. One report indicates the utilization of high-pressure homogenization technology not only to efficiently drive the bacterial membrane to produce bacterial biomimetic vesicles (BBVs) but also to develop a “ring-like” BBV loaded with polymerized RBD (RBD-BBV). The subcutaneous injection of RBD-BBV can lead to accumulation in the lymph nodes of mice, enhancing antigen uptake and processing while inducing specific humoral and cellular immune responses against SARS-CoV-2 [23]. However, it remains uncertain whether BBVs can provide immune protection against APEC in chickens.

In this study, we prepared bacterial membrane vesicles through genetic modification and high-pressure homogenization, compared the protective efficacy of two immunization approaches, namely nasal drops and intramuscular injection, and evaluated the potential of BBVs as a vaccine candidate in specific pathogen-free (SPF) chickens.

## 2. Results

### 2.1. ΔtolA J11 Mutant Increases OMVs Production

**Figure 1:**
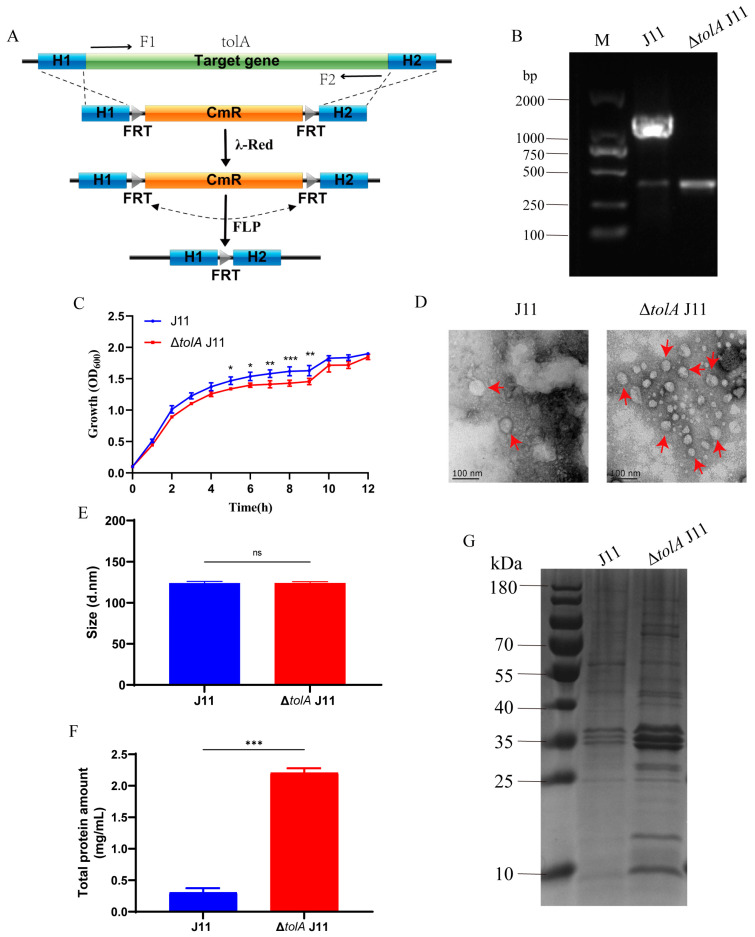
The *tolA* gene mutation in *E. coli* J11 strain increases the total protein content of its released OMVs. (**A**) A schematic diagram of *tolA* gene deletion strain constructed by the λ-Red homologous recombination method; (**B**) the *tolA* mutant was verified by PCR amplification with the size of the *tolA* gene being 1266 bp; (**C**) the growth curve of the mutant and wild-type strains; (**D**) morphological characteristics of vesicles observed by transmission electron microscopy (red arrow); (**E**) particle sizes of vesicles; (**F**) determination of the total protein amount by the BCA method; (**G**) SDS-PAGE analysis (0.5 μg/well). Data were representative of three independent experiments and displayed the mean ± SD. ns: no significance, * *p* < 0.05, ** *p* < 0.01, *** *p* < 0.001.

In pursuit of enhancing OMVs production, the low-pathogenic *E. coli* serotype O78 J11 strain was selected as parent strain and a ΔtolA J11 mutant strain with deletion of the tolA gene using the λ-Red homologous recombination technique was constructed (Figure 1A). Verification of PCR amplification was conducted utilizing tolA gene primers, followed by sequencing analysis. The ΔtolA J11 strain exhibited no amplification of the tolA gene, confirming the successful construction of the gene mutant strain (Figure 1B). The deletion of the tolA gene had an impact on bacterial growth. The OD_600_ value of the ΔtolA J11 strain in bacterial culture significantly decreased during the logarithmic growth phase, and there was no significant difference in the OD_600_ value after a 10 h incubation period when compared to the wild-type strain J11 (Figure 1C). In addition, transmission electron microscopy (TEM) observation showed that the OMVs of both the ΔtolA J11 and J11 strains were spherical nanoparticles, and the number of vesicles of ΔtolA J11 was more than that of J11 (Figure 1D). Particle size analysis showed that the particle size of OMVs of J11 was 123.80 ± 2.23 nm and that of ΔtolA J11 was 123.97 ± 1.72 nm, and there was no significant difference between them (Figure 1E). Due to surface tension effects, the particle size obtained from the analyzer was slightly larger than that determined by TEM. The BCA method was employed to quantify the total protein concentration of the isolated OMVs. It was found that ΔtolA J11 exhibited a significantly higher total protein concentration of 2.21 ± 0.071 mg/L compared to only 0.31 ± 0.067 mg/L for the wild-type strain J11, representing a sevenfold increase (Figure 1F). Furthermore, SDS-PAGE analysis conducted on the isolated OMVs demonstrated the presence of three predominant outer membrane proteins at approximately 35 kDa (Figure 1G). Collectively, these findings indicate that mutation of the tolA gene in *E. coli* J11 strain significantly enhanced the total protein content of its released OMVs.

### 2.2. Production of BBVs Using High-Pressure Homogenization Technology

**Figure 2:**
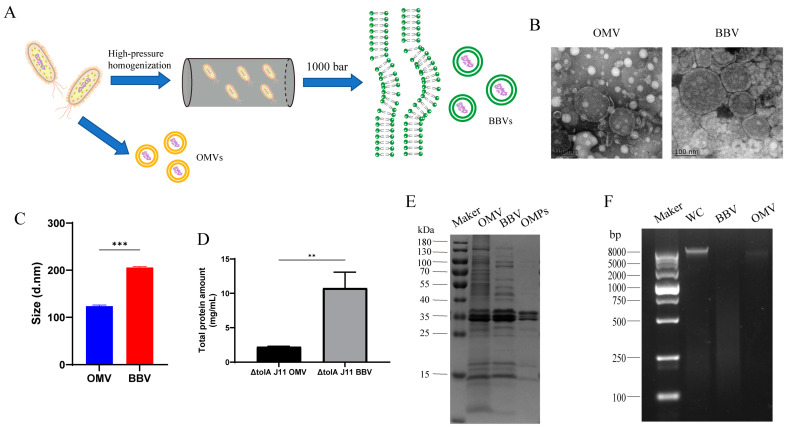
Production of BBV by high-pressure homogenization technology. (**A**) Schematic diagram of bacterial membrane vesicle production; (**B**) electron microscopy analysis of OMVs and BBVs; (**C**) particle size analysis of OMVs and BBVs; (**D**) determination of total protein concentration by BCA assay; (**E**) SDS-PAGE analysis (0.5 μg/well); (**F**): DNA content in samples of Δ*tolA* J11 whole cells (WC), BBV, and OMV were analyzed by agarose gel electrophoresis. Data are representative of three independent experiments and displayed as mean ± SD. ** *p* < 0.01, *** *p* < 0.001.

To further enhance the yield of vesicles, BBVs were prepared by means of high-pressure homogenization technology (Figure 2A). Under electron microscope observation, BBVs presented a complete spherical vesicle structure with an average particle size of 205.77 ± 1.76 nm (Figure 2B,C). Importantly, the total protein yield of artificially driven BBVs was sixfold higher than that of spontaneously generated OMVs (Figure 2D). SDS-PAGE analysis found that both OMVs and BBVs contained three main bands related to outer membrane proteins (Figure 2E). In addition, almost no bacterial nucleic acid was detected in BBVs (Figure 2F), which greatly improved its safety as a vaccine candidate.

### 2.3. OMVs and BBVs Stimulate HD11 to Induce Innate Immune Response

**Figure 3:**
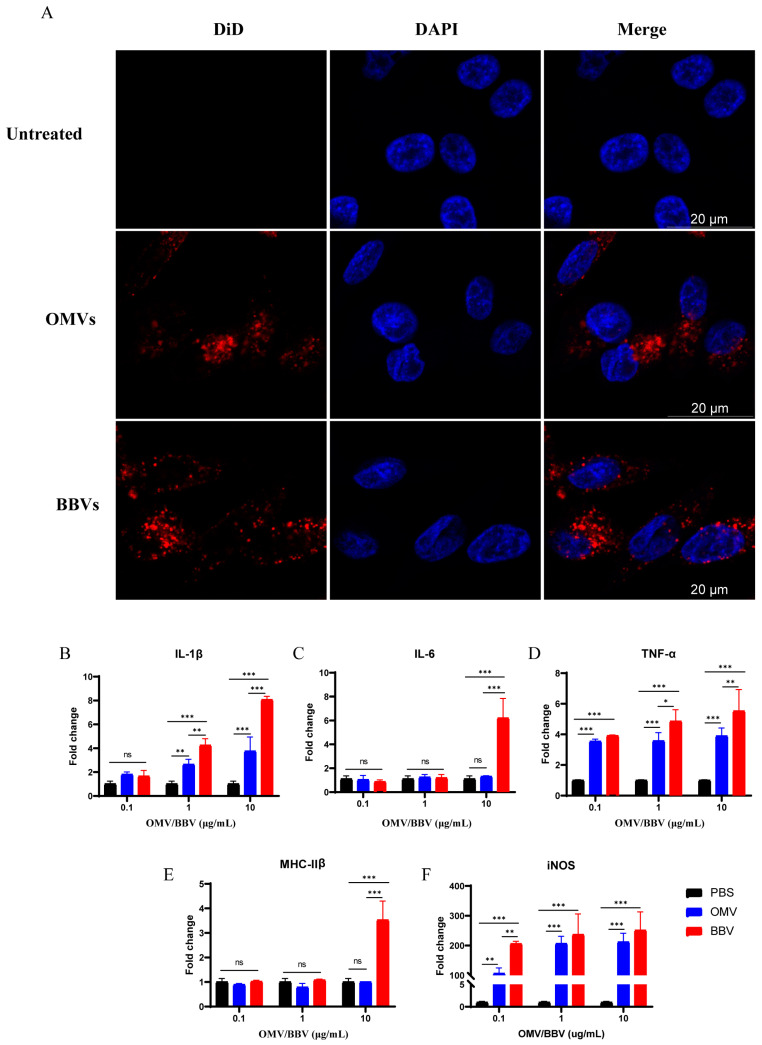
HD11 cells recognize and internalize OMVs and BBVs. (**A**) Confocal microscope observation: OMVs and BBVs were stained with DiD (red), and nuclei were stained with DAPI (blue). mRNA expression of IL-1β (**B**), IL-6 (**C**), TNF-α (**D**), MHC-IIβ (**E**), and iNOS (**F**) in HD11 cells stimulated by OMVs and BBVs. Data were representative of three independent experiments and displayed mean ± SD. ns: no significance, * *p* < 0.05, ** *p* < 0.01, *** *p* < 0.001.

To evaluate whether the OMVs and BBVs could activate the immune response of HD11 cells in vitro, OMVs and BBVs were stained with the red fluorescent lipophilic compound DiD, and the nuclei were labeled with DAPI. HD11 cells were incubated with OMVs and BBVs at 37 °C for 4 h, and then observed by confocal microscopy. The red signals appeared in the cytoplasm of HD11, indicating that HD11 can recognize and take up OMVs and BBVs (Figure 3A). In addition, both OMVs and BBVs stimulated the mRNA expression of pro-inflammatory cytokines (IL-1β, TNF-α, and iNOS) in HD11 cells in a dose-dependent manner, and the expression level induced by BBVs was higher than that induced by OMVs (Figure 3B–F). These results suggest that both OMVs and BBVs can induce innate immune responses in chicken macrophages.

### 2.4. Impacts of OMV and BBV Vaccination on Acquired Immune Response in Chickens

#### 2.4.1. OMVs and BBVs Induce Specific Mucosal Immune and Humoral Immunity Response

**Figure 4:**
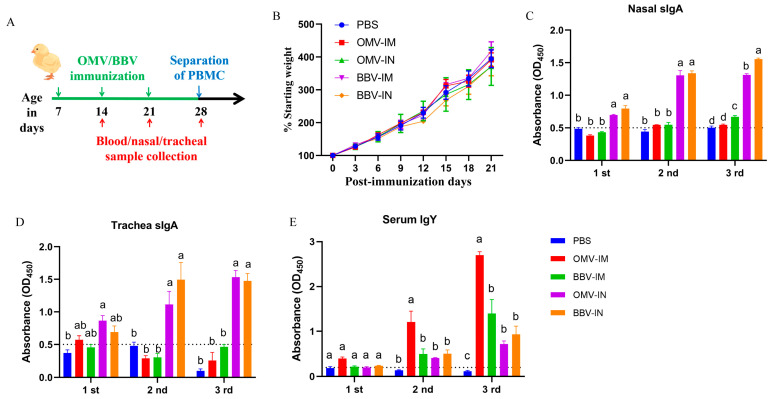
Antibody response induced by intranasal (IN) or intramuscular (IM) immunization with OMVs or BBVs in chickens. (**A**) Schematic of chicken immunization experiments. (**B**) Body weight gain curves of chickens after immunization. C&D: Omps-specific sIgA antibodies in nasal cavity (**C**) and trachea (**D**) of immunized chickens detected by enzyme-linked immunosorbent assay (*n* = 3). (**E**) Omps-specific IgY antibodies in serum samples of immunized chickens detected by enzyme-linked immunosorbent assay (*n* = 5). The 1st, 2nd, and 3rd referred to samples were collected at the first, second, and third post-immunizations, respectively. The data are represented as the mean ± SD. Difference lowercase letters denote statistically significant differences (*p* < 0.05).

To investigate the potential of OMVs and BBVs in eliciting adaptive immune responses, chickens were immunized weekly via intramuscular or intranasal inoculation for three times (Figure 4A). The immunization did not impact the weight gain of the chickens, indicating that immune stress has no influence on the growth performance of chickens (Figure 4B). The antibody levels of Omps-specific sIgA in the nasal cavity and trachea of chickens immunized with OMVs and BBVs, as well as the Omps-specific IgY in the serum, were determined. Following three intranasal immunizations with OMVs or BBVs, OMP-specific sIgA antibodies were significantly increased in the nasal cavity and trachea samples. Conversely, no specific sIgA response was observed in the intramuscular immunization groups or non-immunized control groups (Figure 4C,D). The antibody levels of OMP-specific IgY in the serum samples increased proportionally with the frequency of immunization. In comparison to intranasal immunization, intramuscular immunization with OMVs elicited a rapid and higher titer response of OMP-specific IgY in the serum samples (Figure 4E). The results indicate that intranasal immunization with OMVs or BBVs can induce Omps-specific IgY and sIgA antibodies, thereby establishing mucosal immunity and humoral immunity in vivo.

#### 2.4.2. OMVs and BBVs Induce Specific Cellular Immune Response

**Figure 5:**
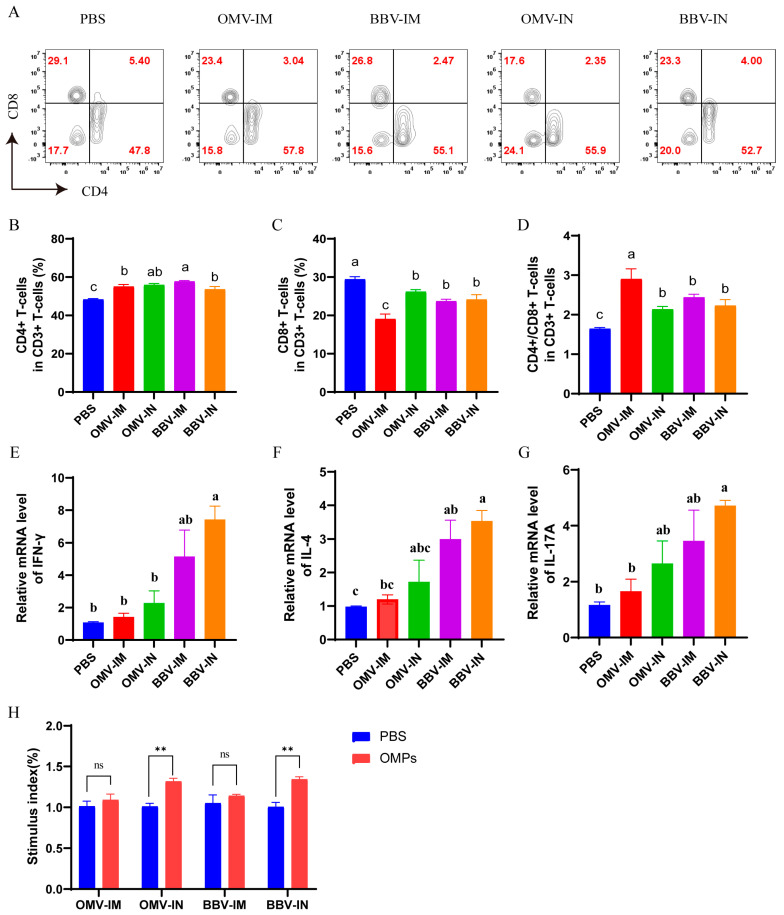
Cellular immune response induced by intranasal (IN) or intramuscular (IM) immunization with OMVs or BBVs in chickens. Representative flow cytometry plots (**A**) and corresponding statistical analysis (**B**–**D**) illustrating proportions of CD4^+^ and CD8^+^ T lymphocytes in peripheral blood lymphocytes of immunized chickens. Additionally, mRNA expression levels of IFN−γ (**E**), IL−4 (**F**), and IL−17A (**G**) in the PBMC were assessed. Proliferation response of chicken peripheral blood lymphocytes was evaluated using CCK−8 method. Compared to PBS, ns: no significance, ** *p* < 0.01 (**H**). Data represented as mean ± SD. Difference lowercase letters denoted statistically significant differences (*p* < 0.05).

To evaluate cellular immune response, T lymphocytes in peripheral blood were subjected to flow cytometry analysis. Both OMV and BBV immunization led to an increase in the proportion of CD4^+^ T lymphocytes in peripheral blood, accompanied by a decrease in the proportion of CD8^+^ T lymphocytes, as well as an elevation in the CD4^+^/CD8^+^ ratio, indicating an enhancement of immune activity among CD4^+^ T cells (Figure 5A–D). Additionally, the analysis of cytokine mRNA expression levels in PBMCs revealed that intranasal immunization of BBVs (BBV-IN) elevated the expression levels of IFN-γ, IL-4, and IL-17A, while intramuscular injection of BBVs (BBV-IM) only significantly increased the expression level of IL-4 (Figure 5E–G). Furthermore, the CCK-8 assay was utilized to assess the proliferation of PBMCs in immunized chickens. The results revealed a significant increase in lymphocyte proliferation within the OMV-IN and BBV-IN groups following stimulation with Omps (Figure 5H). The findings suggest that both OMV and BBV vaccinations have the potential to enhance cellular immune function, particularly through intranasal immunization.

### 2.5. Intranasal Immunization of OMVs and BBVs Effectively Improves Protection Against APEC Challenge

**Figure 6:**
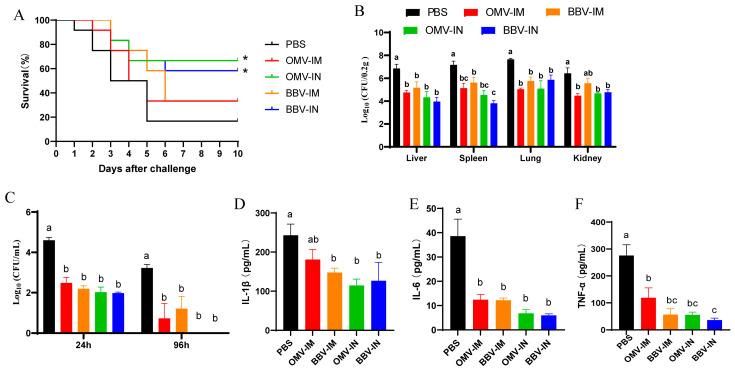
Immunoprotective effects induced by intranasal (IN) or intramuscular (IM) immunization with OMVs or BBVs in chickens. (**A**) Survival rates of immunized chickens following challenge with lethal dose of APEC serotype O78 (*n* = 10). (**B**) Bacterial distribution in various tissues and organs at 24 h post-APEC challenge. (**C**) Bacterial loads in blood at 24 h and 96 h post-APEC challenge. (**D**–**F**) Serum concentrations of IL-1β, IL-6, and TNF-α at 24 h post-APEC challenge. Data represented as mean ± SD. Difference lowercase letters denoted statistically significant differences (*p* < 0.05). * *p* < 0.05.

To assess the potential of OMVs and BBVs as vaccine candidates, chickens were challenged to a lethal dose (10^8^ CFU) of APEC J122 strain, serotype O78, via air sac injection following the completion of the aforementioned immunization protocol, and were monitored for a period of 10 days. Unimmunized chickens (PBS group) began to exhibit mortality within 24 h post-infection, resulting in a 10-day survival rate of only 16.67%. In contrast, chickens immunized via intramuscular injection with OMVs and BBVs started dying 48 h post-infection, achieving a survival rate of 33.33% over the same period. Chickens receiving nasal droplet immunization demonstrated higher survival rates: OMV-IN group at 66.67%, BBV-IN group at 58.3% (Figure 6A). The bacterial load in the liver, spleen, lung, and kidney tissues of immunized chickens was significantly lower than that observed in the PBS group at 24 h post-infection (Figure 6B). Furthermore, APEC remained detectable in the blood of PBS group animals at both 24 h and 96 h post-infection while bacterial levels decreased but were not completely cleared from those receiving OMV-IM and BBV-IM treatment; conversely, complete bacterial clearance was achieved in the OM-IN and BBV-IN immunized cohort (Figure 6C). Additionally, the levels of the pro-inflammatory cytokines IL-1β, IL-6, and TNF-α in serum samples collected from infected chickens at 24 h post-infection were detected. The results indicated that cytokine levels in all immunized groups were significantly reduced compared to those observed in the PBS control group.

**Figure 7:**
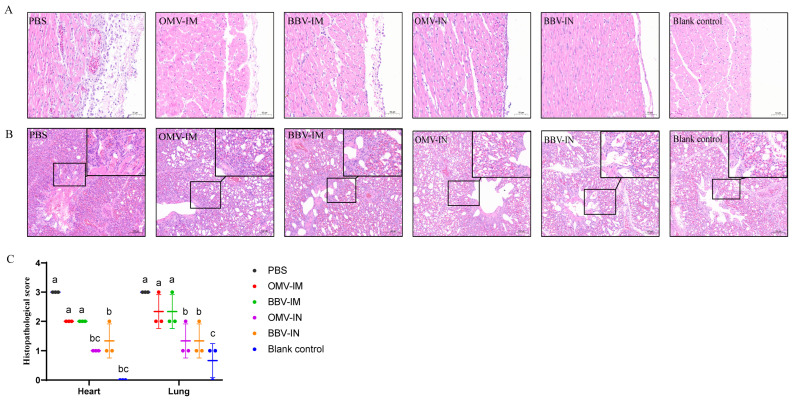
Histopathological lesion in chickens following intranasal (IN) or intramuscular (IM) immunization with OMVs or BBVs and challenge. A&B: Hearts (**A**) and lungs (**B**) were collected from chickens at 3 days post-infection (dpi) and subjected to histological analysis after staining with hematoxylin and eosin (H&E); (**C**): Pathological damage scoring (*n* = 3). Pathological damage was scored on a scale of 0 to 3 (0, not present; 1, slight; 2, moderate; 3, severe). Data represented as mean ± SD. Difference lowercase letters denoted statistically significant differences (*p* < 0.05).

Pathological assessments were performed on the heart and lung tissues of chickens three days after the challenge. Unimmunized chickens (PBS group) exhibited pericardial edema, a heart capsule filled with yellow fibrin exudate, and pericardial adhesions. H&E-stained sections revealed extensive infiltration of inflammatory cells and myocardial congestion (Figure 7A). The lungs displayed severe interstitial pneumonia characterized by pronounced atrial congestion and significant inflammatory cell infiltration. Chickens immunized via intramuscular injection (OMV-IM and BBV-IM) demonstrated moderate pericarditis and interstitial pneumonia. In contrast, those receiving nasal administration (OMV-IN and BBV-IN) showed only mild thickening of the pericardium, with limited inflammatory cell infiltration; similarly, their lung tissues exhibited only mild interstitial pneumonia (Figure 7B). The severity of pathological lesions in both the heart and lungs was assessed using histopathological damage scoring, revealing that intranasally immunized chickens experienced minimal tissue damage to these organs (Figure 7C).

## 3. Discussion

In the production of industrial vaccines, it is necessary to increase the amount of OMVs to minimize the production cost. At present, increasing the yield mainly includes physical, chemical, and genetic engineering methods [18]. Ultrasonic treatment is a common physical method used in OMV production, which leads to the spontaneous assembly of artificial OMVs after bacteria are broken. However, it is difficult for this artificial OMVs to maintain a complete vesicle structure, and its composition is very different from that of spontaneous OMVs [24]. In addition, adding hydrogen peroxide or consuming cysteine can induce oxidative stress and lead to excessive vesiculation of some bacteria [25]. Moreover, gene knockout can increase OMVs production by targeting key genes involved in OMVs biogenesis [26]. In this study, we improved the production of membrane vesicles in two steps: A high-yield vesicle mutant Δ*tolA* J11 was obtained by gene knockout, which resulted in a sevenfold increase in vesicle yield compared to the wild-type strain. Then, BBVs were produced by high-pressure homogenization technology, resulting in a sixfold increase in vesicle yield, finally leading to a remarkable a forty-twofold enhancement compared to the wild-type strain. By adjusting the parameters of the high-pressure homogenizer, the wall-breaking effect can be controlled to ensure the structural integrity of BBVs. The same approach could potentially be employed to enhance the production of OMVs in different bacterial species.

Assessment of vaccine efficacy and safety as critical indicators for evaluating vaccine candidates is critical. Previous studies have indicated that the *tolA* mutant exhibited attenuated virulence in mouse infection models [27]. The vesicle donor strain J11 utilized in this study was an O78 serotype low-pathogenic *E. coli*. Based on this, the deletion of the *tolA* gene might further reduce the toxicity of the strain. Additionally, OMVs containing bacterial nucleic acids pose potential threats, restricting their development as vaccine candidates. In contrast to OMVs, BBVs have a lower degree of nucleic acid contamination. Throughout the entire immunization process, no detrimental effects on the weight gain of chickens were detected, suggesting that BBVs have fewer adverse reactions as vaccine candidates. Additionally, a study utilizing nitrogen cavitation to produce double-layered membrane vesicles (DMVs) demonstrated that *Pseudomonas aeruginosa* (*P. aeruginosa*)-derived DMVs conferred enhanced protection against bacterial infection. Administration of a lethal dose of live *P. aeruginosa* to mice as a form of *P. aeruginosa*-derived OMV immunization extended the mouse life to 48 h. However, *P. aeruginosa*-derived DMVs allowed 50% survival among the mice [28]. In our study, nasal drop immunization with BBVs and OMVs elicited comparable immune responses and protective effects, resulting in only 16.67% survival in unimmunized chickens compared to 66.67% in the OMV-IN group and 58.3% in the BBV-IN group. Therefore, both BBVs and OMVs effectively increased the survival rate of chickens through nasal drip immunization.

Vaccination is intricately associated with the activation of adaptive immunity. It has been demonstrated that most Omps function as protective antigens, stimulating robust T cell-dependent humoral immune responses [29]. Among the Omps derived from APEC OMVs, key components include OmpA, OmpC, and OmpF. Notably, both OmpA and OmpC are ubiquitous across all *E. coli* strains. Thus, these Omps may serve as primary constituents of OMVs that elicit cross-protection [30]. Our study demonstrates that BBVs significantly enhanced the production of Omps, resulting in markedly increased levels of serum-specific IgY directed against Omps, as well as nasal and tracheal specific sIgA. Consequently, Omps may represent a vital immunoprotective component within BBVs. Furthermore, T cell immune responses play a critical role in host defense against bacterial infections [31]. CD4^+^ T cells exhibit differentiation potential into distinct subsets including Th1, Th2, and Th17 cells which secrete IFN-γ, IL-4, and IL-17 cytokines, respectively [32]. The IFN-γ-mediated Th1 response combats intracellular bacteria by enhancing phagocytic bactericidal activity while IL-17-mediated Th17 immunity targets extracellular bacteria through promoting neutrophil recruitment to infection sites [33,34]. We found that both OMVs and BBVs effectively activate CD4^+^ T cells during the vaccination processes, thereby facilitating the activation of helper T cell functions related to Th1 and Th17 pathways. Furthermore, OMVs and BBVs enhance the proliferative capacity of PBMCs via intranasal immunization.

Optimal routes of vaccine administration can enhance or modify the immune response of the body, resulting in diverse protective mechanisms. sIgA, a critical component of mucosal immunity, serves as the primary defense against pathogenic microbial infections and plays an essential role in protecting mucosal surfaces from pathogens [35,36]. Previous studies showed that intranasal immunization with NTHi OMVs elicited a robust and complex humoral and mucosal immune response that effectively prevents challenges by both homologous and heterologous NTHi strains [37]. *Salmonella* OMVs expressing *Streptococcus pneumoniae* (*S. pneumoniae*) proteins induced significant immunoprotection against *S. pneumoniae*-infected mice; notably, a reduction in bacterial recovery from the nasal cavity correlated with localized production of antigen-specific IL-17A [38]. Intranasal immunization significantly reduced airway colonization and prevented systemic bacterial dissemination. In contrast, intramuscular immunization with OMVs elicited an antibody response but was ineffective in preventing *Acinetobacter baumannii* infection [21]. Thus, local mucosal immunization is crucial for preventing and controlling respiratory pathogens. Our study demonstrated that intranasal immunization elicited localized mucosal immune responses in both the nasal cavity and trachea, resulting in a significant increase in specific sIgA levels. This response was associated with a reduction in bacterial load within chickens and an improvement in their survival rate. In contrast, while intramuscular immunization could induce antibody production, it proved ineffective in preventing APEC infection. Therefore, mucosal immunity offered effective protection against APEC.

In conclusion, highly productive BBVs are prepared by genetic modification and high-pressure homogenization technology, and both BBVs and OMVs effectively activate mucosal immunity in the nasal cavity and trachea while simultaneously eliciting both humoral and cellular immune responses in chickens. Nasal administration is identified as the most effective immunological strategy for combating APEC, providing protection to chickens against infection with the O78 serotype of APEC. However, further investigation is required to assess its protective efficacy against other serotypes of APEC. Our research establishes a robust technical foundation for the development of bacterial membrane vesicle vaccine candidate targeting APEC.

## 4. Materials and Methods

### 4.1. Plasmids, Strains, and Cells

Plasmids pKD46, pKD3, and pCP20, which are necessary for λ-Red homologous recombination, were generously provided by Professor Murphy from the University of Massachusetts Medical School, USA, and were maintained in our laboratory [39]. The low-pathogenic *E. coli* J11 and high-pathogenic *E. coli* J122, belonging to the O78 serotype, were isolated from clinical chicken strains and cultured in Luria–Bertani (LB) medium at 37 °C with agitation at 220 rpm. The pathogenicity test of the two strains on 1-day-old specific pathogen-free (SPF) chicks is presented in Figure S1. Chicken macrophage HD11 cells were cultured in 1640 medium supplemented with 10% fetal bovine serum and 0.5% antibiotics (Gibco, Waltham, MA, USA).

### 4.2. Construction of tolA Gene Mutant

The Tol-Pal system is indispensable for maintaining the outer membrane integrity of *E. coli*. To increase the formation of OMVs, we used λ-Red homologous recombination to disrupt the *tolA* gene [39]. The upstream flanking region of the *tolA* gene served as homology arm H1, while the downstream flanking region functioned as homology arm H2. The primer sequences employed for PCR amplification are detailed in Table 1. Initially, chloramphenicol resistance genes, encompassing homology arms H1 and H2, were amplified using the pKD3 plasmid as a template and *tolA* D1/D2 as primers. Subsequently, the J11 strain carrying the pKD46 plasmid was cultivated in LB medium supplemented with ampicillin (50 µg/mL) and 0.2% L-arabinose until OD_600_ = 0.5 at 30 °C. The bacterial cells were then collected and washed three times with ice-cold 10% glycerol to generate electrocompetent cells. The PCR product was introduced into these cells via electroporation (Eppendorf, Hamburg, GER), followed by selection of CmR transformants at 37 °C. After preparing the transformants for electrocompetence, the pCP20 plasmid was introduced into them through electroporation as well. AmR transformants were selected at 30 °C and subsequently incubated at 43 °C under agitation at a speed of 220 rpm, to facilitate loss of the pCP20 plasmid and obtain a *tolA* deletion strain devoid of resistance. The concentration of chloramphenicol is 25 µg/mL, and that of ampicillin is 50 µg/mL. The mutants were confirmed by PCR using internal region primers (*tolA*F1/F2) and sequenced by Tsingke Biotech (Beijing, China).

### 4.3. Production of Outer Membrane Vesicles

OMVs were isolated from bacterial culture supernatants using a previously established method [46]. Briefly, bacterial culture supernatants collected during logarithmic growth phases underwent a series of centrifugation steps. Initially, they were filtered through a 0.45 μm filter and concentrated using a 100 kDa ultrafiltration tube. The resulting concentrated supernatant was then subjected to ultracentrifugation (150,000× *g*, 3 h, 4 °C). The resulting precipitate was resuspended in sterile phosphate-buffered saline (PBS) at pH 7.4, constituting the enriched OMV fraction, which was subsequently stored at −70 °C.

### 4.4. High-Pressure Homogenization Technique to Induce BBVs Production

Bacterial cultures in the logarithmic growth phase were collected following established protocols [23]. The bacterial precipitate was obtained through high-speed centrifugation at 8000× *g* for 10 min, followed by washing with sterile PBS and subsequent resuspension. Cell disruption was achieved using a high-pressure homogenizer (AH-E, Suzhou, China) operating at 1000 bar and 4 °C. The resulting homogenized samples underwent centrifugation at 6000× *g* for 30 min at 4 °C to eliminate impurities, yielding a supernatant. This supernatant was then filtered through a 0.45 μm membrane and concentrated using a 100 kDa ultrafiltration tube. Ultracentrifugation (150,000× *g*, 1 h, 4 °C) was performed to further purify the sample, with the resulting precipitate resuspended in sterile PBS at pH 7.4 to obtain the enriched BBVs. Finally, samples were stored at −70 °C.

### 4.5. Extraction of Outer Membrane Proteins

OMPs were isolated by the Sarkosyl method with slight modifications [47]. Bacterial cultures in the logarithmic growth phase were collected and centrifuged at 10,000× *g* for 20 min at 4 °C to obtain the bacterial precipitate. The precipitate was washed once with pre-cooled Tris-Mg buffer (10 mM Tris-Cl, 5 mM MgCl_2_, pH 7.3), and subsequently resuspended in the same buffer. Ultrasound was employed to fragment the bacteria, followed by centrifugation at 3000× *g* for 10 min at 25 °C to eliminate unfragmented bacteria. The resulting supernatant was carefully aspirated and subjected to ultracentrifugation at 100,000× *g* for 1 h at 4 °C. The precipitate was collected, resuspended in Tris-Mg buffer containing 2% (*w*/*v*) sodium dodecyl sarcosinate (SLS), and incubated at room temperature for 30 min. Subsequently, another round of ultracentrifugation was performed at 70,000× *g* for 60 min at 4 °C to remove cytoplasmic membranes, and the resulting supernatant was discarded. The precipitate containing outer membrane proteins was then resuspended in sterile double-distilled water (ddH_2_O) and stored at −70 °C.

### 4.6. Characterization of Bacterial Membrane Vesicles

Transmission electron microscopy (TEM) visualization was conducted following established protocols [48]. Briefly, samples were adhered to a 300 mesh copper grid and negatively stained with 1% phosphotungstic acid for 30 min. The morphology and integrity of the vesicles were observed using a Tecnai 12 transmission electron microscope (Philips, Amsterdam, NL). The vesicles, at a concentration of 0.5 mg/mL, were homogeneously suspended in PBS buffer (pH 7.4) prior to conducting particle size analysis using the Malvern Particle Analyzer (ES90 Nano, Malvern, UK), with data evaluation performed utilizing Malvern Zetasizer Software (v8.01, Malvern, UK). The protein concentration of the vesicles was determined using a BCA Protein Concentration Assay Kit (Beyotime, Shanghai, China) following the manufacturer’s instructions. For proteomic analysis, proteins were separated by 12% SDS-PAGE gel and stained with Coomassie Brilliant Blue G250 (Thermo Pierce, Waltham, MA, USA). Residual nucleic acids constitute an important quality control criterion for biological products. To detect residual bacterial genomic DNA in vesicles, sample nucleic acids were extracted using the Bacterial Genomic DNA Extraction Kit (Tiangen, Beijing, China), mixed with electrophoresis loading buffer (TaKaRa, Beijing, China), and subjected to 1% agarose gel electrophoresis at 160 V for 30 min. Gel images were acquired and analyzed using a gel imaging system (Bio-Rad).

### 4.7. In Vitro Study of Chicken Macrophages

OMVs and BBVs were labeled with 10 μM Far-red Plasma Membrane Fluorescent Probe (DiD, Beyotime, Shanghai, China) and stained for 20 min on ice in the dark. The mixture was then centrifuged in a 100 kDa ultrafiltration tube and washed three times with PBS to remove residual DiD. HD11 cells (2 × 10^5^ cells/well) were cultured overnight in complete PRMI-1640 medium (Gibco, Waltham, MA, USA) supplemented with 10% heat-inactivated fetal bovine serum (Gibco, Waltham, MA, USA). Following this, they were co-cultured with DiD-labeled OMVs and BBVs (protein concentrations of 0.1, 1, and 10 μg/mL) for 12 h. Subsequently, the cells were washed with PBS, and the nuclei were stained with 4,6-diamidino-2-phenylindole (DAPI, Sigma, St. Louis, Missouri, USA) for 15 min at 37 °C before observation under an ultra-high-resolution laser confocal microscope (Leica, Wetzlar, Germany).

To investigate the immunostimulatory effects of OMVs and BBVs, HD11 cells were co-cultured with DiD-labeled OMVs and BBVs (protein concentrations of 0.1, 1, and 10 μg/mL) for 18 h. PBS-treated cells were employed as a control. The mRNA expression of IL-1β, IL-6, TNF-α, MHC-IIβ, and iNOS in HD11 cells stimulated by OMVs and BBVs were detected by the dye-based SYBR Green qPCR. The cell cultures were collected, and total RNA was extracted using RNA isolater Total RNA Extraction Reagent (Vazyme, Nanjing, China). The extracted RNA was then reverse-transcribed to cDNA using TransScript All-in-One First-Strand cDNA Synthesis SuperMix for qPCR (TRANS, Beijing, China), followed by qPCR (Bioer technology, Hangzhou, China) using PerfectStart Green qPCR SuperMix according to the manufacturer’s instructions. Thermal cycling parameters included pre-denaturation at 94 °C for 30 s, followed by 45 amplification cycles at 94 °C for 5 s and 60 °C for 30 s. The melting curves are shown in Figure S2.

β-actin served as the housekeeping gene. Relative mRNA expression was calculated using the 2^−ΔΔCt^ method and expressed as the fold-change relative to the control, which was normalized to 1 [49]. The primer sequences used in this study are provided in Table 1.

### 4.8. Immunization and Challenge of Chickens

To evaluate the immunoprotective effects of OMVs and BBVs, 7-day-old SPF chickens were randomly divided into five groups (*n* = 12/group), including the OMV intranasal immunity group (OMV-IN), the OMV intramuscular injection group (OMV-IM), the BBV intranasal immunity group (BBV-IN), the BBV intramuscular injection group (BBV-IM), and the control group (PBS). OMVs and BBVs from the same production batch were immunized by nasal drops (50 μg) and intramuscular injection (100 μg), respectively, followed by two booster immunizations at the 14th and 21st day of age, while the control group was injected with PBS only. The mental state, feed intake, and behavioral manifestations of the chickens were observed throughout the immunization process, and the variations in body weights were continuously recorded. Samples, including serum, tracheal, and nasal lavage fluids, were collected one week after the first, second, and third immunizations (1st, 2nd, and 3rd), respectively. Specific IgY antibodies against Omps in serum and specific secretory IgA (sIgA) antibodies against Omps in nasal and tracheal lavage fluids were detected by indirect ELISA. One week after the third immunization, three chickens were randomly selected from each group, and peripheral blood mononuclear cells (PBMCs) were isolated for flow cytometry analysis and lymphocyte proliferation assay. The expression levels of IFN-γ, IL-17A, and IL-4 mRNA in PBMCs were evaluated by qRT-PCR. The primer sequences are presented in Table 1.

Following the vaccination, the O78 serotype APEC J122 strain was inoculated via the air sac route at a dose of 10^8^ CFU. The survival rates were monitored for 10 consecutive days post-challenge. A total of 24 h after bacterial exposure, three chicks per group were randomly euthanized, and their liver, spleen, lung, and kidney tissues were collected for colony counting after grinding with sterile PBS. Blood samples were drawn at 24 and 96 h post-challenge to determine the bacterial load. Serial 10-fold dilutions of blood in sterile PBS were plated on MacConkey’s medium for colony counting. The O antigen serotype of the bacteria on MacConkey agar was identified by PCR to verify that the colony was the strain used (Figure S3). Additionally, serum was collected 24 h after bacterial challenge, and the levels of chicken IL-1β, IL-6, and TNF-α were measured using ELISA kits (Solarbio, Beijing, China) following the manufacturer’s instructions. At 3 days post-infection (dpi), heart and lung tissues were collected, fixed with 4% formaldehyde, made into paraffin sections, and stained with hematoxylin–eosin (Hematoxylin-Eosin, HE) for histopathological examination via microscopy. The histopathological damage was evaluated by two veterinary pathologists. In brief, the lesion scoring was based on a scale of 0–3 (normal = 0, mild = 1, moderate = 2, severe = 3). Finally, the chickens were subjected to cervical dislocation for euthanasia, packaged in sealed bags, frozen for preservation, and collectively sent to the animal carcass harmless treatment center located in Yangzhou University.

### 4.9. Indirect ELISA Assays

Serum antibody levels specific to IgY against Omps and specific to secretory IgA (sIgA) in nasal and tracheal lavage fluids were determined using a previously described indirect ELISA method [50]. Enzyme plates were initially incubated overnight at 4 °C with 50 ng Omps in 100 μL of coating buffer (15.9 mM Na_2_CO_3_, 29.7 mM NaHCO_3_, pH 9.6). Following this, the plates underwent three washes for 10 min each with phosphate-buffered saline solution containing 0.05% Tween 20 (PBST). Subsequently, the plates were blocked with PBST containing 5% skimmed milk for 2 h at 37 °C. Serum samples were then diluted to 1:6400, while nasal and tracheal lavage solutions were diluted 1:5. Next, 100 μL of each diluted sample was added to respective wells, and the plates were incubated at 37 °C for 1 h. The plates were then incubated with 100 μL of rabbit anti-chicken IgY-HRP (H+L) antibody or rabbit anti-chicken sIgA-HRP (H+L) antibody (1:8000 dilution) at 37 °C for 1 h. Following incubation, 100 μL of TMB (Solarbio, Beijing, China) was added to each well, and the samples were left to react for 15 min under light exposure. The reaction was terminated by adding 50 μL of 2M H_2_SO_4_, and the optical density (OD) was measured at 450 nm in an automatic microplate reader.

### 4.10. Flow Cytometry Analysis

PBMCs from immunized chickens were isolated using the Chicken PBMC Isolation Kit (Solarbio, Beijing, China), labeled with murine anti-chicken CD3-FITC, CD4-PE, and CD8a-APC antibodies (Southern Biotech, Birmingham, AL, USA), and incubated for 30 min in the dark. The PBMCs were then washed with PBS to remove unbound antibodies, and the proportions of CD3^+^ T cells, CD4^+^ T cells, and CD8^+^ T cells were analyzed by flow cytometry (BD Accuri C6, USA).

### 4.11. Lymphocyte Proliferation Assay

Cell proliferation was assessed using the CCK-8 assay as previously described [51]. PBMCs from immunized chickens were isolated using the Chicken PBMC Isolation Kit (Solarbio, Beijing, China). After counting, 106 cells per well were seeded in 96-well plates containing RPMI 1640 medium supplemented with 10% fetal bovine serum and stimulated with Omps at a concentration of 20 μg. The cells were then cultured at 37 °C with 5% CO_2_ for 48 h. Subsequently, each well was incubated with 10 μL of CCK-8 solution (Beyotime, Shanghai, China) for 1 h at 37 °C in the dark, and the optical density (OD) was measured at 450 nm using an enzyme marker. The stimulation index (SI) was calculated as the OD of OMP-stimulated cells divided by the OD of unstimulated cells.

### 4.12. Statistical Methods

Data calculations were performed using graphpad Prism 9.0 software. The data are expressed as means ± the standard deviation (SD). Pairwise comparisons were made using Student’s *t*-test. Differences greater than two groups were analyzed by one-way ANOVA analysis with the Newman–Keuls post-test, with *p* < 0.05 as the level of statistically significant differences.

## Figures and Tables

**Table 1:** Primers used in this study.

Gene	Sense Primer (5′–3′)	References
*tolA* F1	GTGTCAAAGGCAACCGAACAAA	This study
*tolA* F2	TTACGGTTTGAAGTCCAATGGCG	
*tolA* D1	GAGAGCGGGTAACAGGCGAACAGTTTTTGGAAACCGAGAgtgtaggctggagctgcttc	This study
*tolA* D2	GGTGCCTGATGTTGACCGTCCGAACAGTCAACATCGCGAtggtccatatgaatatcctccttagttcc	
IL-1β F	TCGACATCAACCAGAAGTGC	[40]
IL-1β R	GAGCTTGTAGCCCTTGATGC	
IL-6 F	CAAGGTGACGGAGGAGGAC	[41]
IL-6 R	TGGCGAGGAGGGATTTCT	
TNF-α F	ACAGGACAGCCTATGCCAAC	[40]
TNF-α R	ACAGGAAGGGCAACACATCT	
iNOS F	AGGCCAAACATCCTGGAGGTC	[42]
iNOS R	TCATAGAGACGCTGCTGCCAG	
MHC-IIβ F	GTGCAGAGGAGCGTGGAG	[43]
MHC-IIβ R	CGTTCAGGAACCACTTCACC	
β-actin F	GAGAAATTGTGCGTGACATCA	[44]
β-actin R	CCTGAACCTCTCATTGCCA	
IL-4 F	TGAATGACATCCAGGGAGAG	[45]
IL-4 R	GGCTTTGCATAAGAGCTCAG	
IFN-γ F	AGCTGACGGTGGACCTATTATT	[45]
IFN-γ R	CTGCAGATCATCCACCGGAA	
IL-17A F	CTCCGATCCCTTATTCTCCTC	[45]
IL-17A R	AAGCGGTTGTGGTCCTCAT	

## Data Availability

The data will be made available upon request.

## Supplementary Materials

The following supporting information can be downloaded at: https://www.mdpi.com/article/10.3390/ijms252212055/s1.
