# Supplementary material for: Development of High-Production Bacterial Biomimetic Vesicles for Inducing Mucosal Immunity Against Avian Pathogenic Escherichia coli
Source: Int J Mol Sci. 2024 Nov 9;25(22):12055. doi: 10.3390/ijms252212055 (PMC11593933; doi:10.3390/ijms252212055)
Supplement: Supplementary file 1 [file ijms-25-12055-s001.zip › ijms-3278477-supplementary.pdf]

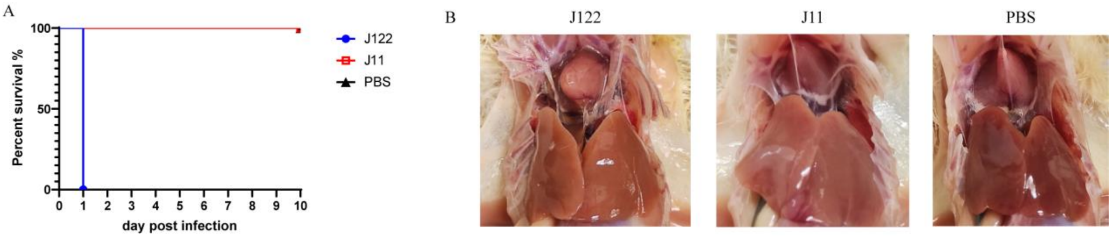

Figure S1. Pathogenicity testing of strains. A: Survival rate after challenge. 1-day-old SPF chickens were injected with 10^7 CFU of J122 or J11 strains through the left thoracic air sac and continuously observed for 10 days; B: Clinical lesions. The heart of the J122 strain showed pericardial effusion, while no obvious lesions were found in the J11 and PBS.

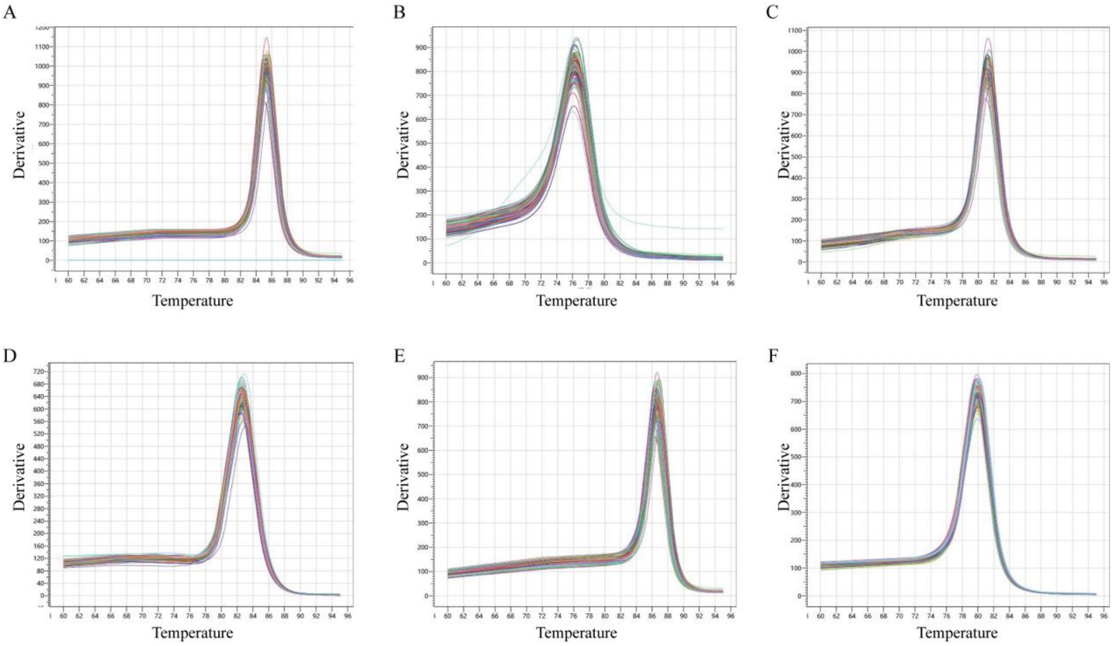

Figure S2. Melting curve. During qPCR amplification, melting profiles were obtained for the β-actin (A), IL-1β (B), IL-6 (C), TNF-α (D), MHC-IIβ (E) and iNOS (F) genes.

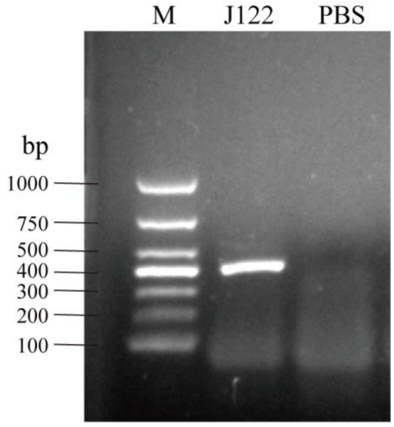

Figure S3. PCR verification of strains. The O antigen serotype of the strains (J122) isolated from challenged chickens was confirmed through PCR. M: DL 1000 DNA maker, O78: 399bp.
